# Single-Cell Transcriptomics-Based Study of Transcriptional Regulatory Features in the Mouse Brain Vasculature

**DOI:** 10.1155/2021/7643209

**Published:** 2021-07-23

**Authors:** Wei-Wei Lin, Lin-Tao Xu, Yi-Sheng Chen, Ken Go, Chenyu Sun, Yong-Jian Zhu

**Affiliations:** ^1^Department of Neurosurgery, Second Affiliated Hospital of Zhejiang University School of Medicine, Zhejiang University, 88 Jiefang Road, Hangzhou, 310009 Zhejiang, China; ^2^Department of Orthopedics, Shanghai General Hospital, Shanghai Jiao Tong University School of Medicine, Shanghai Jiao Tong University, 100 Haining Road, Shanghai 200080, China; ^3^Clinical Training Centre, St. Marianna Hospital, Japan; ^4^Internal Medicine, AMITA Health Saint Joseph Hospital Chicago, 2900 N. Lake Shore Drive, Chicago, 60657 Illinois, USA

## Abstract

**Background:**

The critical role of vascular health on brain function has received much attention in recent years. At the single-cell level, studies on the developmental processes of cerebral vascular growth are still relatively few. Techniques for constructing gene regulatory networks (GRNs) based on single-cell transcriptome expression data have made significant progress in recent years. Herein, we constructed a single-cell transcriptional regulatory network of mouse cerebrovascular cells.

**Methods:**

The single-cell RNA-seq dataset of mouse brain vessels was downloaded from GEO (GSE98816). This cell clustering was annotated separately using singleR and CellMarker. We then used a modified version of the SCENIC method to construct GRNs. Next, we used a mouse version of SEEK to assess whether genes in the regulon were coexpressed. Finally, regulatory module analysis was performed to complete the cell type relationship quantification.

**Results:**

Single-cell RNA-seq data were used to analyze the heterogeneity of mouse cerebrovascular cells, whereby four cell types including endothelial cells, fibroblasts, microglia, and oligodendrocytes were defined. These subpopulations of cells and marker genes together characterize the molecular profile of mouse cerebrovascular cells. Through these signatures, key transcriptional regulators that maintain cell identity were identified. Our findings identified genes like Lmo2, which play an important role in endothelial cells. The same cell type, for instance, fibroblasts, was found to have different regulatory networks, which may influence the functional characteristics of local tissues.

**Conclusions:**

In this study, a transcriptional regulatory network based on single-cell analysis was constructed. Additionally, the study identified and profiled mouse cerebrovascular cells using single-cell transcriptome data as well as defined TFs that affect the regulatory network of the mouse brain vasculature.

## 1. Introduction

The critical role of vascular health on brain function has received much attention in recent years [1]. There is a close correlation between the expression of cerebrovascular-specific genes and neurovascular-related diseases. On the other hand, the blood-brain barrier (BBB) is a unique feature of the cerebrovascular system, and it is necessary for the functioning of the nervous system. By developing tissue-specific properties, the vascular system forms a selective BBB that allows passage of essential molecules to the brain and locks the penetration of potentially harmful compounds or cells. Nonetheless, BBB may be a key barrier to the treatment of brain diseases as revealed in humans and animal models [2, 3]. Research into the characteristics of vascular cells is critical and advances diagnostic and therapeutic techniques for the cerebrovascular system [1]. Even so, the transcriptional regulatory features between cerebrovascular cells remain unclear.

Transcription factors (TFs) have long been recognized as important aspects in the maintenance of cellular identity and function [4]. Increased or decreased TF expression can significantly affect cellular function and can recode cells into different cell types [5–7]. However, the process of cerebral vascular growth and development, at the single-cell level, is still poorly studied. For example, the ability of vascular smooth muscle cells (VSMCs) to contract is critical to the regulation of blood pressure and flow. Nonetheless, there is a lack of prior research on the transcriptional regulation of VSMC contractile function at the individual cell level [8]. Significant progress has been made in recent years in the construction of gene regulatory networks (GRNs) based on single-cell transcriptome expression data [4, 9]. With advances in single-cell sequencing, we can begin to understand the transcriptional regulatory networks in cells.

In this study, we utilized a comprehensive atlas of obtained mouse cerebrovascular single-cell data [1] to construct a single-cell transcriptional regulatory network of mouse cerebrovascular cells. To realize this, the study used single-cell transcriptome data in conjunction with the GRN approach. In the study, we initially defined the TFs that affect the regulatory network of the mouse brain vasculature where it was revealed that even similar cell types have different regulatory networks.

## 2. Materials and Methods

### 2.1. Datasets

A single-cell RNA-seq dataset of mouse brain vasculature was downloaded from GEO (GSE98816) [1]. For each batch of cells, we calculated the number of genes expressed per cell. Genes that expressed less than 0.1% of the cell count were excluded from the study. Such batch of cells did not contain the mitochondrial gene. Ultimately, all cells in this dataset met quality control criteria, and a total of 3186 cells were included for analysis.

### 2.2. Dimensional Reduction and Clustering

Principal component analysis (PCA) together with JackStraw and PCEIbowPlot functions was performed using the Seurat package (version 3.2.2), in R software (version 4.0.2), to select important principal components (PCs) [10, 11]. Seurat's FindAllMarkers function was used to identify specific genes for each cell subpopulation. The RunUMAP function was then used for cell clustering and visual analysis of UMAP. The marker genes were thereafter annotated with the singleR package and corrected with CellMarker according to their characteristics [12, 13].

### 2.3. Inference of Regulons and Activity

A number of methods have been developed to predict GRNs from single-cell gene expression data. This study adapted the SCENIC method as previously described with slight modification [9, 14]. In the SCENIC analysis process, three steps were considered. First, there was the establishment of a gene coexpression network through gene coexpression analysis. Second, we established possible TF-target regulatory relationships based on the gene coexpression network. In this step, the direct regulatory relationship was established using motif analysis. Any direct downstream genes occurring for each TF were profiled as regulon. It is important to note that, currently, SCENIC only supports transcriptional positive regulation analysis. Third, based on the results of step 2, a regulon activity score (RAS) was calculated for each cell. As described in previous studies, the Avg20 method was repeated three times to assess the variability of random sampling. Thereafter, a *t*-test was used to assess whether the Avg20 method performed better than using all individual cells [4].

### 2.4. Functional Validation

As in previous studies, we used SEEK analysis to verify whether the predicted regulons correlated with their cell type [15]. In brief, we used the mouse version of SEEK to assess whether genes in the regulons were coexpressed. Significantly coexpressed genes in multiple datasets associated with a particular cell type scored positive for high relevance of the function of the regulon to that cell type.

### 2.5. Regulon Module Analysis and Quantifying Cell Type Relationship

To identify regulon modules, we employed two main steps [4, 16]. First, each pair of regulatory relationships was analyzed for Pearson's correlation coefficient. The activity score of each regulon module in relation to a cell type was then defined as the average of the activity scores of its regulon members in all cells of that cell type. The highest ranked units were then filtered for each module. We quantified the relationship between different cell types based on the similarity of overall regulon activity. A pair of cell types was linked if their Spearman correlation coefficient was greater than 0.8. Finally, we used the Markov Clustering Algorithm (MCL) to identify related cell types [17].

## 3. Result

### 3.1. Cell Heterogeneity in the Brain Vasculature

**Figure 1:**
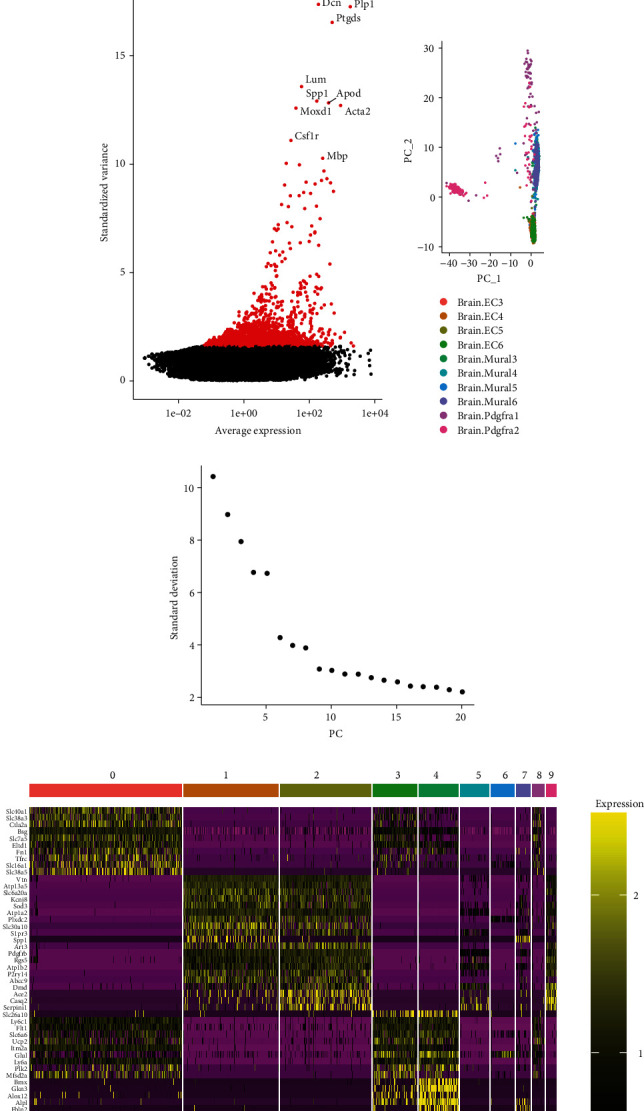
RNA-seq data revealed cell heterogeneity in the brain vasculature. (a) Quality control of 10 copies of cells from mouse brain vasculature, resulting in the inclusion of 3186 cells; (b) ANOVA analysis demonstrates the correlation of the expression of genes in mouse brain vasculature cells. Highly variable genes are indicated by red dots, and nonvariable genes are indicated by black dots. The most variable genes (Dcn, Plp1, Ptgds, Lum, Spp1, Apod, Moxd1, Acta2, Csf1r, and Mbp) are indicated in the figure; (c) analysis with PCA shows that no significant separation of brain vasculature cells occurred in mice; (d) PCA identified 10 PCs as the best differentiation; (e) a heat map showing the top 10 marker genes for each cell cluster.

Ten copies of cells from mouse brain vasculature were checked for quality control (GSE98816), and the resultant 3186 cells were included in the study (Figure 1(a)). The correlation of gene expression in the mouse cerebrovascular cells using ANOVA revealed Lum, Spp1, Apod, Moxd1, Acta2, Csf1r, and Mbp as the most variable genes (Figure 1(b)). Analysis with PCA (PC 1 and PC 2) showed that there was no significant separation of mouse cerebrovascular cells (Figure 1(c)). As shown in Figure 1(d), the model having the best clustering results of 10 PCs was selected. The heat map showed that it identified the 10 most important genes in each of these 10 clusters (Figure 1(e)).

### 3.2. Cellular Subpopulation Distribution and Marker Genes in Mouse Cerebrovascular Cells

**Figure 2:**
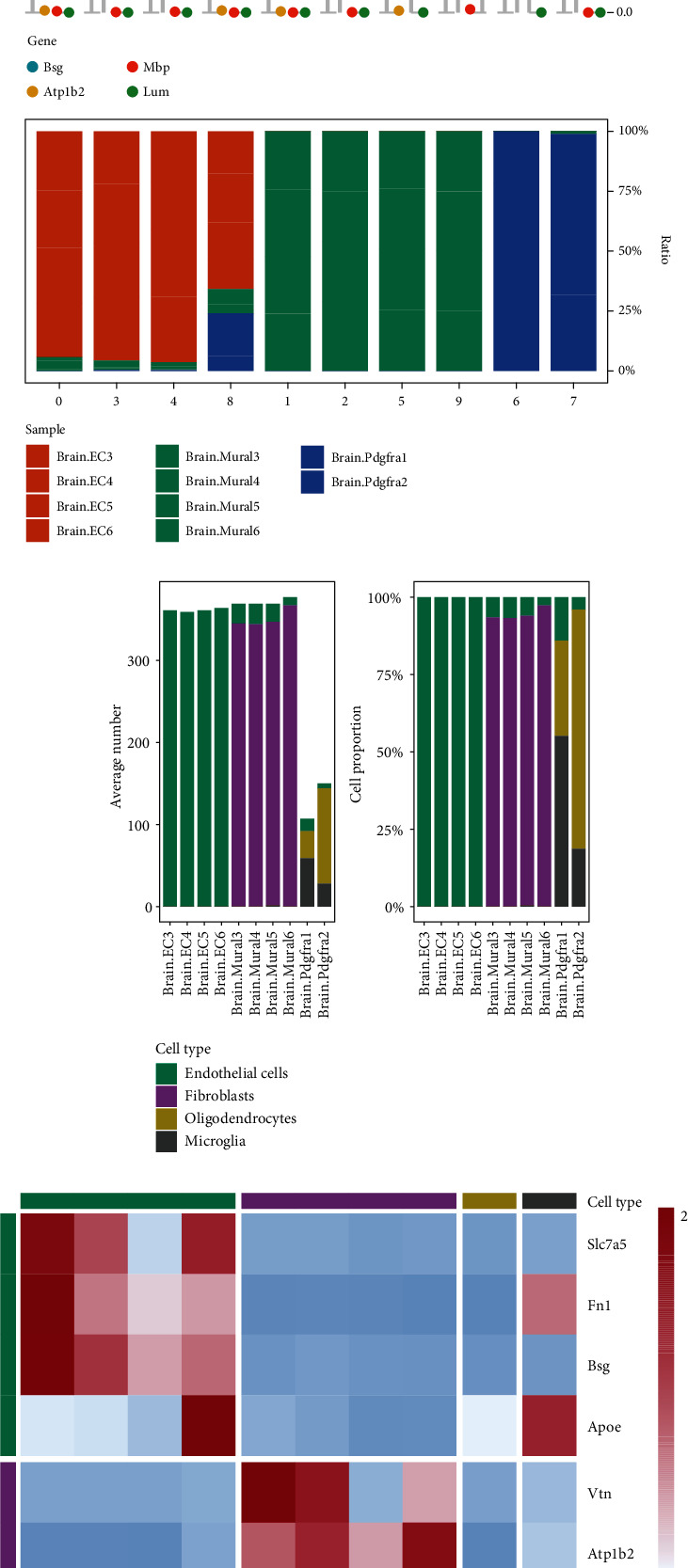
Tumor endothelial cells, fibroblasts promoting angiogenesis, and tissue remodeling. (a) UMAP and cell annotation figures showing mouse brain vasculature cells, colour coded with cell clusters and cell subpopulations (endothelial cells, fibroblasts, oligodendrocytes, and microglia). (b) Sample preference of each cluster. (c) Mean cell numbers and relative proportions of subpopulations of mouse brain vasculature cells derived from each tissue. Brain EC, *n* = 4 samples; brain mural, *n* = 4 samples; brain Pdgfra, *n* = 2 samples. (d) Heat map showing selected mouse brain vasculature cells in each cell cluster. The relative expression profiles of marker genes associated with each cell subpopulation are known. The average expression values are adjusted by averaging and converted from high to low on a scale from -2 to 2 according to expression.

The nine cell clusters were annotated separately using singleR and CellMarker according to the expression profile of the marker genes. Mouse cerebrovascular cells showed 10 clusters (Figure 2(a)). The majority of cerebrovascular cell clusters observed belonged to normal tissue and known vascular cell types such as endothelial cells, fibroblasts, oligodendrocytes, and microglia (Figure 2(a)). Four major marker genes, namely, Bsg, Atp1b2, Mbp, and Lum, distinguished these four cell types as shown in Figure 2(b). Significant differences were seen in the mean cell numbers and relative proportions of subpopulations of mouse cerebrovascular cells derived from each tissue (Figures 2(b) and 2(c)). Marker genes for endothelial cells mainly included Slc7a5, Fn1, Apoe, and Bsg. The main marker genes for fibroblasts included Vtn, Atp1b2, Fos, and Notch3. The main marker genes for oligodendrocytes included Mbp and Cldn11, while those for microglia included Lum and Dcn (Figure 2(d)).

### 3.3. Analysis of Cell Type-Specific Regulation in the Mouse Brain Vasculature

**Figure 3:**
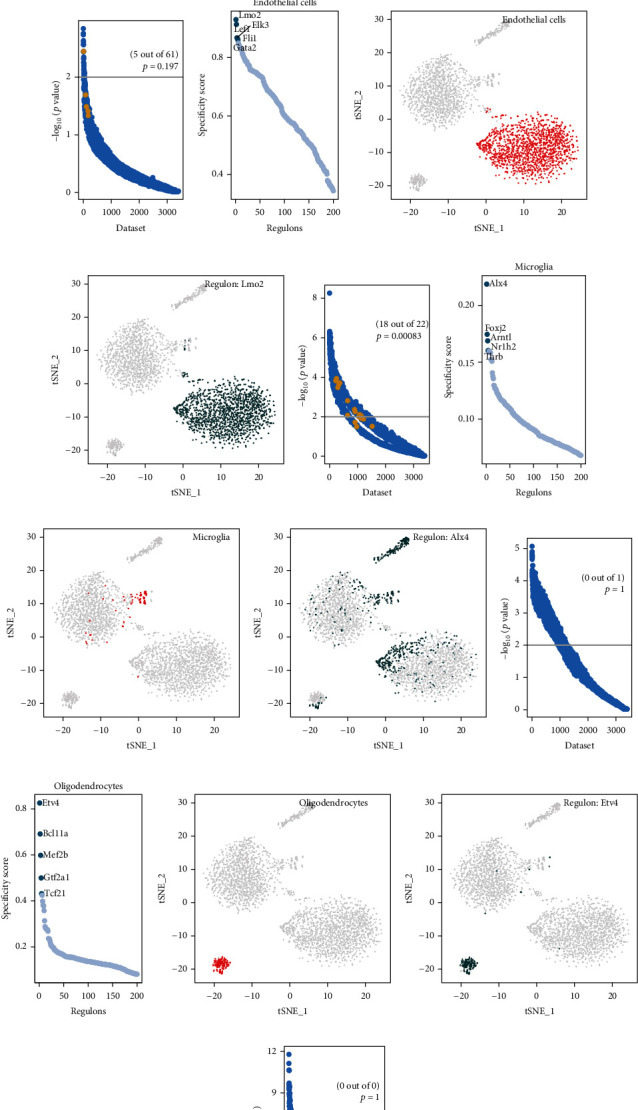
Analysis of cell type-specific regulation in the mouse brain vasculature. (a–d) Fibroblasts: (a) rank for regulons in mouse brain vasculature cells based on regulon specificity scores; (b) endothelial cells are highlighted as red dots in the tSNE plot; (c) the expression values of the genes with the highest regulon activity scores are presented in the tSNE plot; (d) the SEEK analysis is used to find coexpression results of the top regulated genes in different GEO datasets. The *x*-axis represents the different datasets, and the *y*-axis represents the coexpression significance of the target gene in each dataset. Relevant datasets with significant correlation (*p* value < 0.05) are highlighted with yellow dots. (e–h) Same as (a–d) but for endothelial cells. (i–l) Same as (a–d) but for microglia. (m–p) Same as (a–d) but for oligodendrocytes.

We systematically analyzed key transcriptional regulators in each mouse cerebrovascular cell. For each pair of regulatory relationships, we defined a regulon specificity score (RSS) based on the Jensen-Shannon scatter [4, 18]. We then selected the specific regulatory factors with the highest RSS values and further examined their functional properties. Our network analysis identified Cebpa, Zic1, Zfp467, Srebf2, and Mef2c as specific regulators associated with fibroblasts (Figure 3(a)). The tSNE plot further demonstrated that the expression of Cebpa was highly specific in fibroblasts (Figures 3(b) and 3(c)). To test the validity of the above analysis, we applied SEEK analysis to determine GEO datasets with significant coexpression of the regulatory gene Cebpa (Figure 3(d); Fisher's exact test, *p* = 0.197). Using the same approach, we identified Lmo2, Lef1, Elk3, Fli1, and Gata2 as specific regulators associated with endothelial cells (Figure 3(e)). The tSNE plot further demonstrated that the expression of the regulatory factor Lmo2 was highly specific in endothelial cells (Figures 3(f) and 3(g)). To test the validity of the above analysis, we applied SEEK analysis to find GEO datasets that significantly coexpressed the regulatory gene Lmo2, with significant correlation (Figure 3(h); Fisher's exact test, *p* = 0.00083). Second, the most relevant specific regulators of microglia were Alx4, Foxj2, Arntl, Nr1h2, and Thrb (Figure 3(i)). Alx4 expression was not found to be significantly specific in microglia (Figures 3(j) and 3(k)). The most relevant specific regulators of oligodendrocytes were found to be Etv3, Bcl11a, Mef2b, Gtf2a1, and Tcf21 (Figure 3(m)). Etv4 expression was found to be significantly specific in oligodendrocytes (Figures 3(n) and 3(o)). The SEEK analysis did not reveal significant coexpression of the regulatory genes Alx4 and Etv4 in the GEO dataset (Figures 3(l) and 3(p)).

### 3.4. Organizing Regulons into Combinatorial Modules

**Figure 4:**
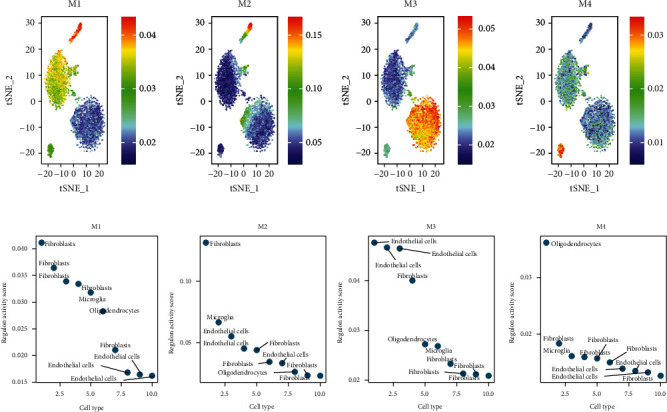
Activity of regulatory modules in different types of mouse brain vasculature cells. (a) Identified regulatory modules (M1-M4) based on the regulatory CSI matrix and mapped average activity of each module onto the tSNE; (b) rank for regulons in mouse brain vasculature cells based on regulon specificity scores. The *y*-axis represents the regulon activity score; the *x*-axis represents the cell type.

**Figure 5:**
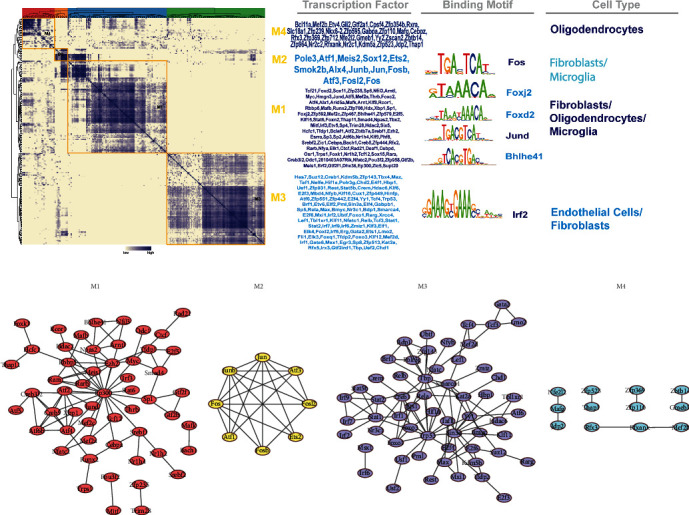
Identification of combinatorial regulon modules. (a) Determination of the regulon modules based on the regulation CSI matrix, along with associated cell types, corresponding binding motifs, and representative transcription factors. (b) Protein-protein interaction networks of regulator factors in each module.

To systematically describe regulatory relationships of TFs, we compared the regulon activity scores of each regulatory relationship pair based on the connection specificity index (CSI) [16]. Thereafter, basing on the regulatory CSI matrix modules identified (M1-M4), we mapped the average activity of each module onto the tSNE (Figure 4(a)). The mouse cerebrovascular cells were then ranked depending on regulon specificity scores (Figure 4(b)). The results showed that each module occupied a different region, with all highlighted regions suggesting the location of high transcriptional activity for different modules (Figure 4(a)). Among them, the M1 and M2 modules showed higher transcriptional activity mainly in fibroblast cells. In addition, the M2 module showed greater specificity. The M3 and M4 modules showed higher transcriptional activity primarily in endothelial cells and in oligodendrocytes, respectively. Gene Ontology (GO) and Kyoto Encyclopedia of Genes and Genomes (KEGG) enrichment analysis was also performed on the differentially expressed genes (DEGs) in each model. Bar graphs of the enrichment analysis were plotted (*p* < 0.05; Supplementary Figures 1 and 2).  Figure 5(a) shows the determination of the regulation module based on the regulation CSI matrix, along with associated cell types, corresponding binding motifs, and representative transcription factors. Interestingly, fibroblasts were found to be involved in all three major modules (M1, M2, and M3). The protein-protein interaction network of regulator factors in each module is shown in Figure 5(b) and Supplementary Figure 3. The M1 module contained the regulatory factors Cebpa, Zic1, Srebf2, and Mef2c, as well as regulator factor Nr1h2, which were transcriptional regulators of fibroblasts and microglia in that order. Alx4 was included in the M2 module, which was a transcriptional regulator of microglia. Lmo2 was included in module M3, which was a transcriptional regulator of endothelial cells. Elsewhere, Etv4, Bcl11a, Mef2b, and Gtf2a1 were included in module M4, which were transcriptional regulators of oligodendrocytes. Combining the results in Figures 3 and 5(a), we speculated that Lmo2 may play an important role in endothelial cells.

## 4. Discussion

In the present study, we used retrieved single-cell RNA-seq to analyze the heterogeneity of mouse cerebrovascular cells, and four cell types (endothelial cells, fibroblasts, microglia, and oligodendrocytes) were defined. Together, these cell subpopulations and marker genes characterize the molecular profile of mouse cerebrovascular cells. Through these features, key transcriptional regulators that maintain cell identity are identified. Our findings reveal that genes including Lmo2 play an important role in endothelial cells.

Significant progress has been made in recent years in the construction of GRNs based on single-cell transcriptome expression data [4, 9]. However, the process of cerebral vascular growth and development at the single-cell level is still poorly studied. In this study, we utilized a comprehensive atlas of obtained mouse cerebrovascular single-cell data [1] and constructed a single-cell transcriptional regulatory network of mouse cerebrovascular cells using single-cell transcriptome data in conjunction with the GRN approach. Four main marker genes distinguished four cell types (endothelial cells, fibroblasts, microglia, and oligodendrocytes): Bsg, Atp1b2, Mbp, and Lum in the present study. There was no significant difference revealed in the mean cell numbers and relative proportions of subpopulations of mouse cerebrovascular cells derived from each tissue. The expression of BSG in endothelial cells has been found to be positively correlated with age in humans, which may explain the increased risk of cardiovascular disease with advancing age [19]. Atp1b2 was found to be associated with changes in the microenvironment within the brain, and we hypothesize that the expression of fibroblasts may affect the microenvironment within the brain [20, 21]. The main marker genes for oligodendrocytes in the present study were Mbp and Cldn11. Similar findings were reported in previous studies [22]. The main marker genes of microglia included Lum and Dcn, both of which have been found to be associated with the development and progression of a variety of tumors [23].

Among the transcription factor regulatory networks, Lmo2 was noted as the most important possible regulator of endothelial cells. The transcriptional regulatory relevance of Lmo2 was significantly defined in 18 datasets (out of 22). In other studies, the transcription factor Lmo2 was found to be an important transcription factor in determining the angiogenic properties of tumors, and it can significantly affect the growth and development of neurovascular cells [24–26]. Several other TFs (Lef1, Elk3, Fli1, and Gata2) have also been found to be associated with the characteristics of endothelial cells. Alx4 has recently been found to be associated with cognitive impairment, congenital disorders of the brain, and normal function of the nervous system [27–29]. However, there are no studies on the interrelationship between microglia and Alx4. In the present study, Alx4 was identified as a potential microglia regulator. The main function of oligodendrocytes in central nervous cells is to provide support and isolation for axons. Although oligodendrocyte development is associated with a variety of factors, its most important regulation is still unknown. Etv4 was identified in the present study as possibly one of the most important regulators of oligodendrocytes. Previous studies have found that mutations in CIC promote malignant progression of gliomas and that Etv4 is implicated in the transcriptional regulation of CIC [30, 31].

Interestingly, fibroblasts were covered in all three main modules (M1, M2, and M3). Crosstalk of fibroblasts in the three modules suggests that they may be important cells affecting the cerebrovascular microenvironment in mice. The transcriptional profile of fibroblasts may also greatly influence the cerebrovascular microenvironment, as has been demonstrated in previous studies [32–35]. In addition, through network analysis, we identified Cebpa, Zic1, Zfp467, Srebf2, and Mef2c as specific regulators associated with fibroblasts. The Cebpa, Zic1, Srebf2, and Mef2c have been found to be associated with the development of fibroblasts in many studies [36, 37]. High expression of ZFP467 was found to be associated with altered vascular morphology and the presence of an inflammatory microenvironment [38].

Knowledge of cellular heterogeneity has greatly increased with the recent availability of single-cell sequencing technology. However, information about mechanisms by which these cellular heterogeneities are established and maintained is rare. The present study provides a new approach to understanding the developmental and functional relationships between vascular cell types in mice. Through the development of a transcriptional regulatory network of major cell types in the mouse brain vasculature, the study further presents protocols and recommendations for prospect studies on neurovascular disease. In the current study, we fully acknowledge that the predicted results remain hypothetical, and further cellular and animal experiments are needed to justify our findings. In addition, prospect studies will need to employ multiple datasets to investigate commonalities between mice and humans to facilitate clinical translation of the research.

## 5. Conclusion

In this study, a transcriptional regulatory network based on single-cell analysis was constructed. In the process, we identified and profiled mouse cerebrovascular cells and incorporated a GRN approach using single-cell transcriptome data. In the study, TFs that affect the regulatory network of the mouse brain vasculature were defined. Further TFs, including Lmo2, which may play an important role in brain endothelial cells, were defined. In addition, we found that even similar cell types have different regulatory networks, which may affect the functional characteristics of local tissues.

## Data Availability

All data used in this paper are from the GSE98816 dataset of the GEO database.

## Abbreviations

BBB: Blood-brain barrier
CSI: Connection specificity index
GRNs: Gene regulatory networks
PCA: Principal component analysis
RSS: Regulon specificity score
RAS: Regulon activity score
SEEK: Search-based Exploration of Expression Kompendia
SCENIC: Single-cell regulatory network inference and clustering.

## Supplementary Materials

Supplementary Materials Supplementary Figure 1: Gene Ontology enrichment pathways of transcription factors in modules M1-M4 (A-D). Supplementary Figure 2: KEGG functional pathways of transcription factors in modules M1-M3 (A-C). Supplementary Figure 3: protein-protein interaction networks of regulator factors in modules M1-M4 (A-D) based on STRING.
