# Supplementary material for: Single-Cell Transcriptomics-Based Study of Transcriptional Regulatory Features in the Mouse Brain Vasculature
Source: Biomed Res Int. 2021 Jul 23;2021:7643209. doi: 10.1155/2021/7643209 (PMC8324343; doi:10.1155/2021/7643209)
Supplement: Supplementary Materials — Supplementary Figure 1: Gene Ontology enrichment pathways of transcription factors in modules M1-M4 (A-D). Supplementary Figure 2: KEGG functional pathways of transcription factors in modules M1-M3 (A-C). Supplementary Figure 3: protein-protein interaction networks of regulator factors in modules M1-M4 (A-D) based on STRING. [file 7643209.f1.docx]

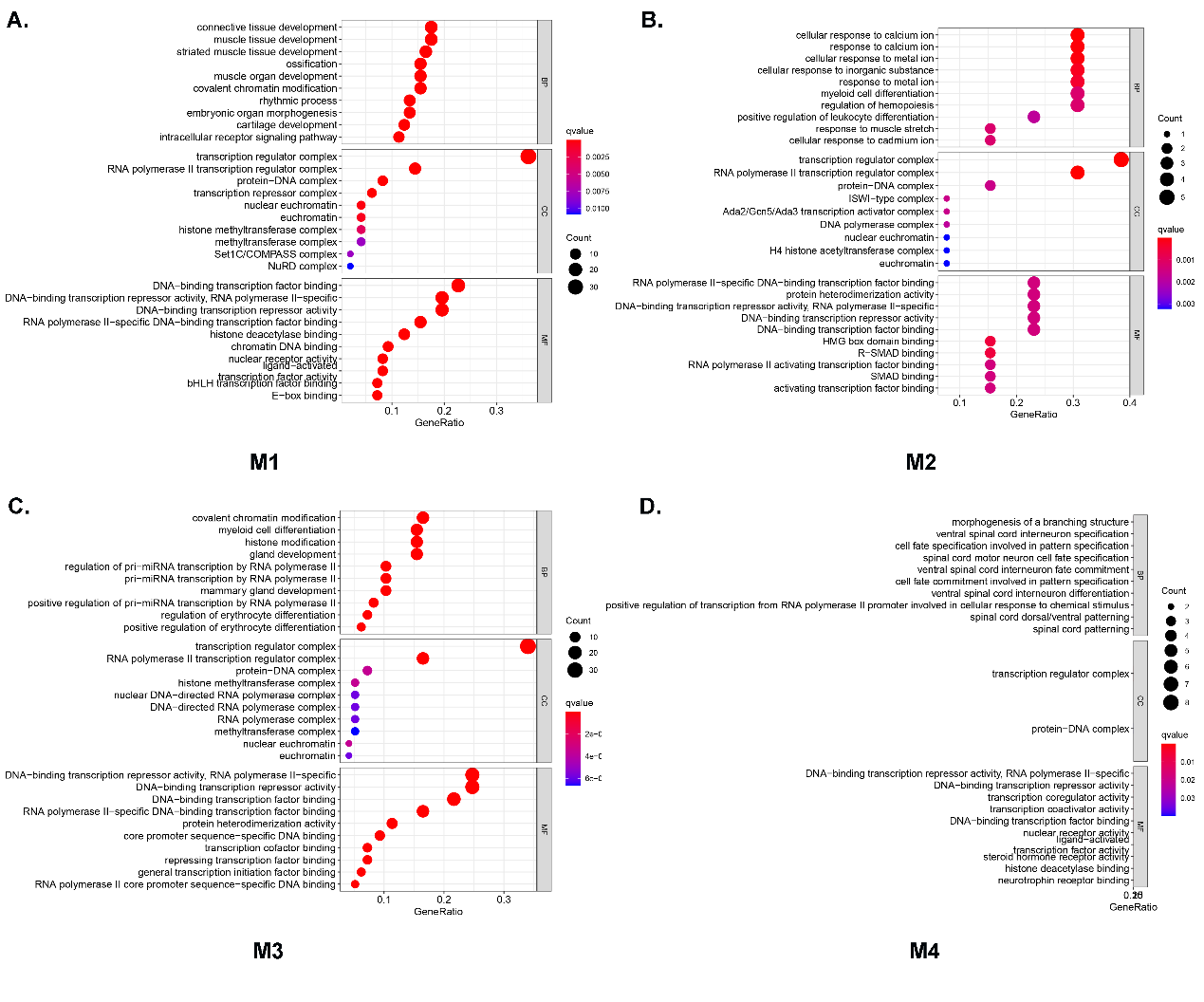


Supplementary figure 1. Gene oncology enrichment pathways of transcription factors in module M1-M4(A-D).


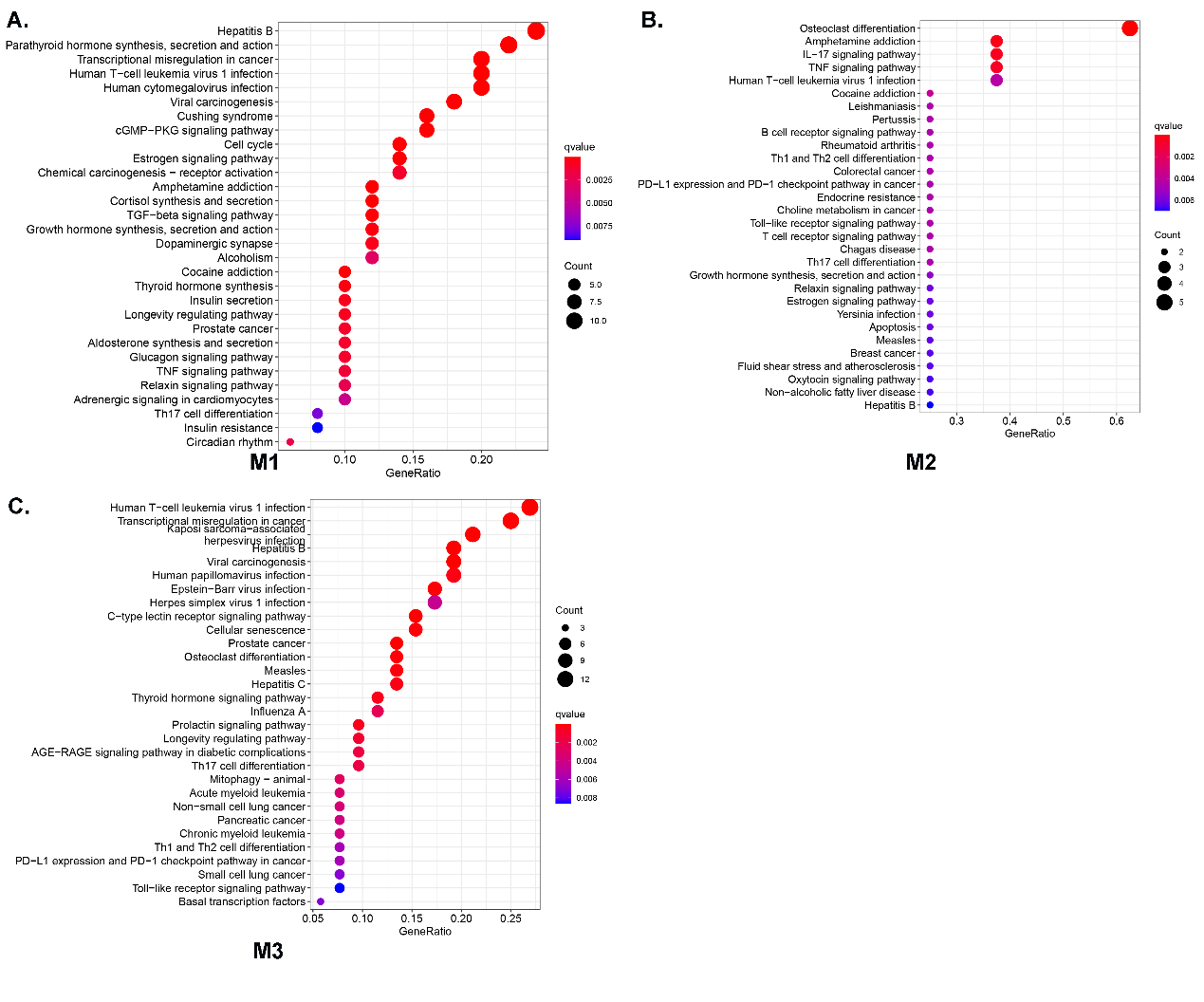


Supplementary figure 2. KEGG functional pathways of transcription factors in module M1-M3(A-C).


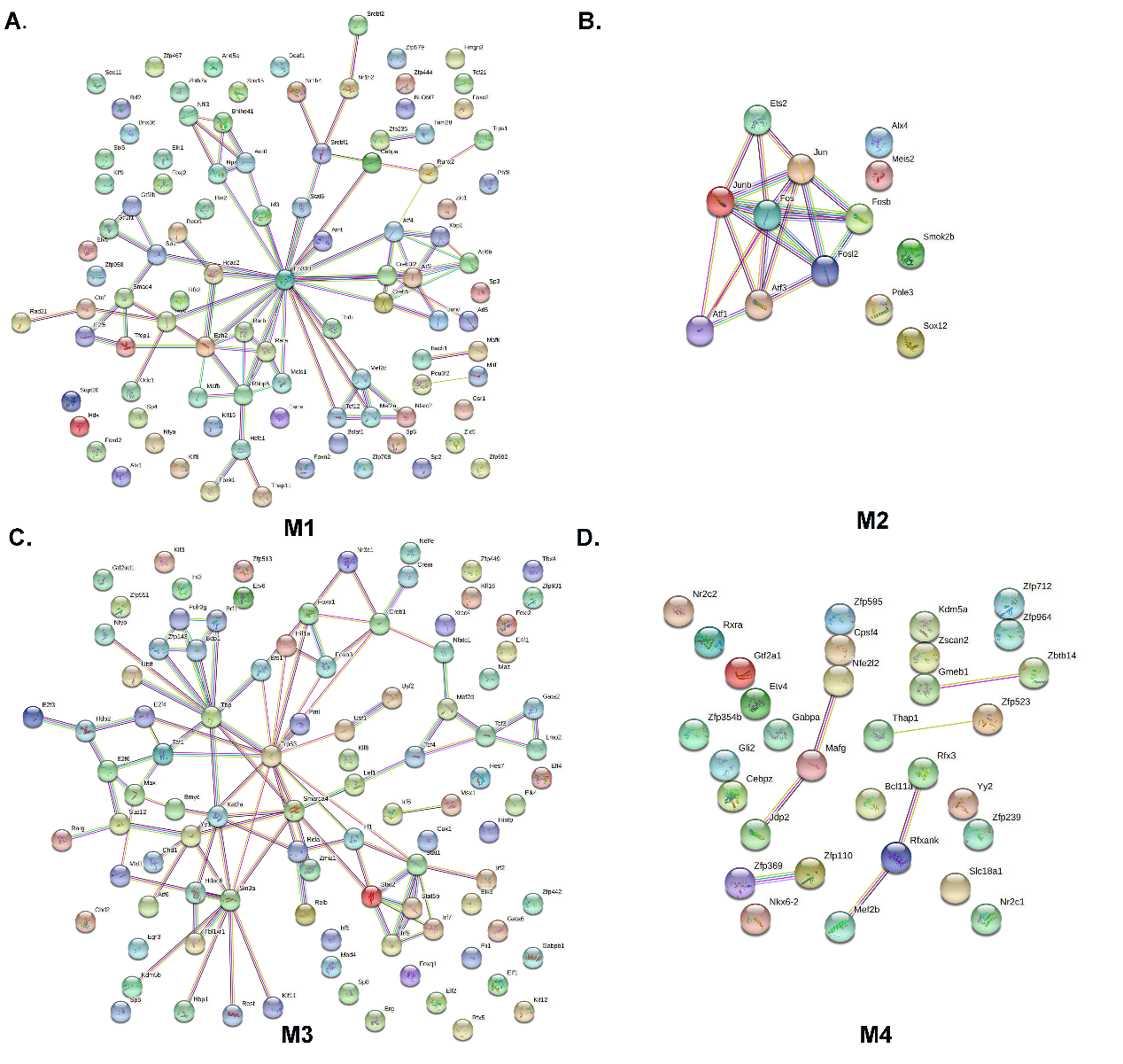


Supplementary figure 3. Protein-protein interaction networks of regulator factors in module M1-M4 (A-D) based on STRING.
